# Draft Genome Sequence of *Bacillus humi* LMG 22167^T^ (DSM 16318), an Endospore-Forming Bacterium Isolated from Soil

**DOI:** 10.1128/genomeA.01692-15

**Published:** 2016-02-04

**Authors:** Jie-ping Wang, Bo Liu, Guo-hong Liu, Zhizhen Pan, Rong-feng Xiao, Meichun Chen, De-ju Chen

**Affiliations:** Agricultural Bio-Resources Research Institute, Fujian Academy of Agricultural Sciences, Fuzhou, Fujian, China

## Abstract

*Bacillus humi* LMG 22167^T^ is a Gram-positive, aerobic, and spore-forming bacterium Here, we report the 4.80-Mb draft genome sequence of *B. humi* LMG 22167^T^, which is the first genome sequence of this species and will promote its fundamental research.

## GENOME ANNOUNCEMENT

The type strain LMG 22167^T^ (=DSM 16318^T^) of *Bacillus humi* was isolated from the soils of the Drentse A agricultural research area in the Netherlands (1). A thermophilic *B. humi* strain could be found by a culture-independent analysis throughout the composting process of swine and mushroom cultural wastes in a field-scale composter (2). *B. humi* was also one of the plant-growth-promoting bacilli from a moderately acidic soil (3). Another thermophilic *B. humi* strain could produce l-lactic acid in a homofermentative manner under high aeration conditions (4). Because of the physiological properties, application prospects, and no available genomic information of *B. humi*, its type strain LMG 22167^T^ was selected as one of the research objects in our genome sequencing project for genomic taxonomy and phylogenomics of *Bacillus*-like bacteria. Here, we present the first draft genome sequence of *B. humi*.

The genome sequence of *B. humi* LMG 22167^T^ was obtained by paired-end sequencing on the Illumina HiSeq 2500 system. One DNA library with an insert size of 239 bp was constructed and sequenced. After filtering of the 0.86-Gb raw data, the 0.81-Gb clean data were obtained, providing approximately 169-fold coverage. The reads were assembled via SOAPdenovo software version 1.05 (5), using a key parameter K setting at 76. Through the data assembly, 50 scaffolds with a total length of 4,795,243 bp were obtained, and the scaffold *N*_50_ was 461,487 bp. The average length of the scaffolds was 95,904 bp, and the longest and shortest scaffolds were 966,545 bp and 523 bp, respectively. A total of 95.10% clean reads could be aligned back to the genome, which covered 98.87% of the sequence.

The annotation of the genome was performed using the NCBI Prokaryotic Genomes Automatic Annotation Pipeline (PGAAP) (http://www.ncbi.nlm.nih.gov/genome/annotation_prok) utilizing GeneMark, Glimmer, and tRNAscan-SE tools (6). A total of 4,973 genes were predicted, including 4,900 coding sequences, 68 tRNAs, and 5 rRNAs. There were 3,490 and 2,181 genes assigned to the COG and KEGG databases, respectively. The average DNA G+C content was 38.15 %, agreeing with the value 37.5 mol% (1).

### Nucleotide sequence accession numbers.

This whole-genome shotgun project has been deposited at DDBJ/EMBL/GenBank under the accession number LMBW00000000. The version described in this paper is the first version, LMBW01000000.

## References

[1] Heyrman J, Rodríguez-Díaz M, Devos J, Felske A, Logan NA, De Vos P 2005 *Bacillus arenosi* sp. nov., *Bacillus arvi* sp. nov. and *Bacillus humi* sp. nov., isolated from soil. Int J Syst Evol Microbiol 55:111–117. doi:10.1099/ijs.0.63240-0. 15653863

[2] Cho KM, Lee SM, Math RK, Islam SM, Kambiranda DM, Kim JM, Yun MG, Cho JJ, Kim JO, Lee YH, Kim H, Yun HD 2008 Culture-independent analysis of microbial succession during composting of swine slurry and mushroom cultural wastes. J Microbiol Biotechnol 18:1874–1883. 19131687

[3] Yadav S, Kaushik R, Saxena AK, Arora DK 2011 Diversity and phylogeny of plant growth-promoting bacilli from moderately acidic soil. J Basic Microbiol 51:98–106. doi:10.1002/jobm.201000098. 21077114

[4] Tongpim S, Meidong R, Poudel P, Yoshino S, Okugawa Y, Tashiro Y, Taniguchi M, Sakai K 2014 Isolation of thermophilic l-lactic acid producing bacteria showing homo-fermentative manner under high aeration condition. J Biosci Bioeng 117:318–324. doi:10.1016/j.jbiosc.2013.08.017. 24119530

[5] Li R, Zhu H, Ruan J, Qian W, Fang X, Shi Z, Li Y, Li S, Shan G, Kristiansen K, Li S, Yang H, Wang J, Wang J 2010 *De novo* assembly of human genomes with massively parallel short read sequencing. Genome Res 20:265–272. doi:10.1101/gr.097261.109. 20019144 PMC2813482

[6] Aziz RK, Bartels D, Best AA, DeJongh M, Disz T, Edwards RA, Formsma K, Gerdes S, Glass EM, Kubal M, Olsen GJ, Olson R, Osterman AL, Overbeek RA, McNeil LK, Paarmann D, Paczian T, Parrello B, Pusch GD, Reich C, Stevens R, Vassieva O, Vonstein V, Wilke A, Zagnitko O 2008 The RAST server: Rapid Annotations using Subsystems Technology. BMC Genomics 9:75. doi:10.1186/1471-2164-9-75. 18261238 PMC2265698

